# HER2 intratumoral heterogeneity predicts response to neoadjuvant therapy in HER2-positive breast cancer: impact and interplay with HER3 expression

**DOI:** 10.1007/s00428-025-04310-3

**Published:** 2025-10-30

**Authors:** Angela Santoro, Federica Vegni, Antonio d’Amati, Francesca Addante, Giuseppe Angelico, Antonio Franco, Alessandra Fabi, Esther Diana Rossi, Gian Franco Zannoni, Antonino Mule

**Affiliations:** 1 Anatomic Pathology Unit, Fondazione Policlinico Universitario Agostino Gemelli IRCCS, Università Cattolica del Sacro Cuore, Rome, 00168 Italy; 2 Department of Translational Biomedicine and Neuroscience, University of Bari Medical School, Bari, 70124 Italy; 3 Department of Medicine and Surgery, Kore University of Enna, Enna, 94100 Italy; 4 Breast Unit, Department of Woman, Child and Public Health, Fondazione Policlinico Universitario Agostino Gemelli IRCCS, Largo Agostino Gemelli, Rome, 00136 Italy; 5 Precision Medicine Unit in Senology, Fondazione Policlinico Universitario A. Gemelli IRCCS, Largo Agostino Gemelli 8, Rome, 00168 Italy

**Keywords:** HER2, HER3, Breast cancer, Intratumoral heterogeneity, Neoadjuvant therapy

## Abstract

Intratumoral heterogeneity (ITH) of HER2 expression and HER3 upregulation have been associated with resistance to HER2-targeted therapies. However, their predictive role in the neoadjuvant setting remains controversial. We retrospectively analyzed 59 patients with HER2-positive invasive breast carcinoma treated with neoadjuvant chemotherapy and anti-HER2 agents at the Agostino Gemelli University Hospital (2018–2020). HER2 ITH was assessed on pre-treatment biopsies and residual tumors (when applicable) by immunohistochemistry and SISH. HER3 expression was also evaluated using IHC and categorized as negative, low, or high. The association with pathological complete response (pCR) and event-free survival (EFS) was assessed. pCR was achieved in 49.2% of patients. HER2 ITH was present in 23.7% of biopsies and was significantly associated with a lower pCR rate (*p* = 0.005). In multivariate analysis, HER2 ITH (OR 0.156, *p* = 0.030), HER2 score (3 + vs 2 + , OR 9.63, *p* = 0.044), and PgR negativity (OR 0.306, *p* = 0.029) emerged as independent predictors of pCR. HER3 expression did not significantly correlate with pCR or EFS, although a non-significant trend toward reduced EFS was observed in HER3-high cases. HER2 ITH negatively impacts pathological response to neoadjuvant therapy in HER2-positive breast cancer and may serve as a potential predictive biomarker. HER3 expression, while not significantly associated with outcome in this cohort, warrants further investigation as a possible contributor to therapeutic resistance. Standardized assessment protocols for both markers could improve patient stratification, guide treatment intensification, and support the integration of novel targeted agents in HER2-positive breast cancer.

## Introduction

Breast cancer is the most commonly diagnosed malignancy in women and a leading cause of cancer-related death worldwide [1]. Among its molecular subtypes, HER2-positive breast cancer—defined by HER2 protein overexpression or ERBB2 gene amplification—accounts for approximately 15–20% of cases and has historically been associated with aggressive clinical behavior and poor outcomes [2]. The advent of HER2-targeted therapies, including trastuzumab, pertuzumab, and more recently antibody–drug conjugates (ADCs), has dramatically improved prognosis, especially in early-stage disease [3].

In the neoadjuvant setting, pathological complete response (pCR) after combined chemotherapy and anti-HER2 therapy is considered a strong surrogate marker for long-term survival. Nonetheless, a significant proportion of HER2-positive tumors do not achieve pCR, underscoring the need for robust biomarkers to guide risk stratification and optimize treatment intensity [4].

Intratumoral heterogeneity (ITH) of HER2 expression, defined by the coexistence of tumor cell subpopulations with different HER2 immunophenotypes or gene copy numbers, has emerged as a critical factor limiting therapeutic efficacy [5, 6]. HER2 ITH has been associated with resistance to both monoclonal antibodies and ADCs, including trastuzumab deruxtecan, particularly when HER2 expression is non-uniform or falls within the "HER2-low" spectrum [7].

HER3 (ERBB3), a kinase-impaired member of the EGFR family, plays a complementary role in resistance mechanisms. By forming heterodimers with HER2, HER3 activates the PI3K/AKT signaling pathway, promoting cell survival and reducing sensitivity to HER2 blockade [8]. Preclinical studies have shown that HER3 upregulation may mediate resistance to trastuzumab, lapatinib, and endocrine therapy [9]. Recently, HER3-targeting agents such as patritumab deruxtecan (HER3-DXd) have shown clinical activity in HER3-expressing metastatic breast cancer, further highlighting the relevance of HER3 in therapeutic resistance and the need to assess its expression in clinical practice [10].

In this retrospective study, we evaluated the predictive and prognostic roles of HER2 intratumoral heterogeneity and HER3 protein expression in a well-characterized cohort of HER2-positive breast cancer patients treated with neoadjuvant chemotherapy and HER2-targeted agents. Our aim was to investigate their association with pCR and event-free survival (EFS), and to explore their utility as biomarkers for resistance and treatment personalization. This study aims to address these gaps by analyzing HER2 ITH and HER3 expression in a real-world cohort treated with standardized neoadjuvant regimens, assessing their predictive and prognostic value.

## Materials and methods

### Study design and patient selection

This retrospective, single-center study was conducted at the Fondazione Policlinico Universitario “Agostino Gemelli” in Rome, Italy. The study was approved by the local ethics committee and conducted in accordance with the Declaration of Helsinki. We reviewed the clinical and pathological records of patients diagnosed with invasive HER2-positive breast carcinoma who underwent neoadjuvant chemotherapy combined with HER2-targeted therapy between July 2018 and December 2020.

Inclusion criteria were: histopathological diagnosis of invasive breast carcinoma with HER2 positivity, defined either as immunohistochemistry (IHC) 3 + or as IHC 2 + with gene amplification confirmed by silver in situ hybridization (SISH); availability of both the pre-treatment core needle biopsy and the post-treatment surgical specimens; and completion of the planned neoadjuvant treatment followed by surgical resection. HER2 positive patients were divided into three groups: ER/PgR neg (absence of hormone receptors); ER/PgR pos (ER and/or PgR receptors greater than 10%) and ER/PgR low (ER and/or PgR receptors between 1 and 9%).

Patients with tumors classified as luminal (ER-/PgR-positive and HER2-negative) or triple-negative breast cancer were excluded, as were patients for whom pre-treatment biopsies or post-surgical material were unavailable.

### Pathological assessment

All pathological assessments were performed on formalin-fixed, paraffin-embedded (FFPE) tissue sections. HER2 expression was evaluated by IHC using standardized protocols, and cases with an equivocal IHC score (2 +) underwent further assessment with dual-probe SISH assays. HER2 positivity was defined according to the ASCO/CAP 2018 guidelines [11].

Intratumoral heterogeneity (ITH) of HER2 was defined as the coexistence of distinct tumor cell populations with different HER2 IHC scores (0, 1 + , 2 + , 3 +) within the same tumor sample. Heterogeneity was documented when significant regional variations were observed under light microscopy, confirmed by repeat IHC and, where appropriate, by SISH.

HER3 expression was assessed by IHC using a validated anti-HER3 monoclonal antibody (D22C5, Cell Signaling Technology, USA). The staining pattern was evaluated semi-quantitatively by assessing the intensity and completeness of membranous staining. HER3 expression was classified as negative (0), low (1 +), or high (2 + or 3 +) based on the proportion and intensity of positive tumor cells, adopting criteria previously proposed by Krop et al. [10].

### Treatment and response evaluation

All patients received neoadjuvant chemotherapy consisting of anthracycline- and taxane-based regimens, combined with trastuzumab, with or without pertuzumab, according to standard institutional protocols. Following the completion of systemic therapy, patients underwent surgical resection of the primary tumor and axillary lymph nodes.

Pathologic complete response (pCR) was defined as the complete absence of residual invasive carcinoma in both the breast and lymph nodes (ypT0/is ypN0), while the presence of residual ductal carcinoma in situ (DCIS) did not preclude the definition of pCR. Surgical specimens were extensively sampled, and histopathological evaluation was conducted according to current recommendations, including hematoxylin and eosin staining and immunohistochemistry where necessary.

### Statistical analysis

Statistical analyses were conducted using SPSS software, version 26.0 (IBM Corp., Armonk, NY, USA). Based on the cohort size and observed pCR rate, the study had approximately 80% power to detect an OR ≥ 2.5 at alpha = 0.05. Continuous variables were expressed as mean ± standard deviation (SD) or median with interquartile range (IQR), depending on data distribution. Categorical variables were presented as absolute numbers and percentages.

Our study aimed to evaluate the possible associations between clinical (age, BMI, clinical T and N stage) and pathological (biological status, HER2 and HER3 expression) variables and the achievement of pCR, with the goal of assessing whether these char-acteristics influenced the achievement of complete response after neoadjuvant chemo-therapy. These associations were assessed using the Fisher’s exact test for categorical variables and the analysis of variance (ANOVA) test for continuous variables. Variables found to be statistically significant for this association were then tested using multivariate logistic regression to identify independent predictors of pCR. Event-free survival (EFS) was estimated using the Kaplan–Meier method, and differences between survival curves were compared using the log-rank test. A two-sided p-value of less than 0.05 was con-sidered statistically significant in all analyses.

## Results

### Patient characteristics

A total of 59 patients with HER2-positive invasive breast carcinoma met the inclusion criteria and were analyzed. The mean age at diagnosis was 51.6 ± 12.3 years, and the mean body mass index (BMI) was 25.5 ± 4.5 kg/m^2^. The majority of tumors were of no special type (NST) histology (83.1%), followed by invasive lobular carcinoma (3.4%) and other special histologies (13.6%). Most tumors were high grade, with 86.4% graded as Nottingham grade 3.

Regarding hormone receptor status, 66.1% of tumors were estrogen receptor (ER) positive and 52.5% were progesterone receptor (PgR) positive. HER2 evaluation revealed that 81.4% of tumors were IHC 3 + , while 18.6% were IHC 2 + with amplification confirmed by SISH.

**Fig. 1:**
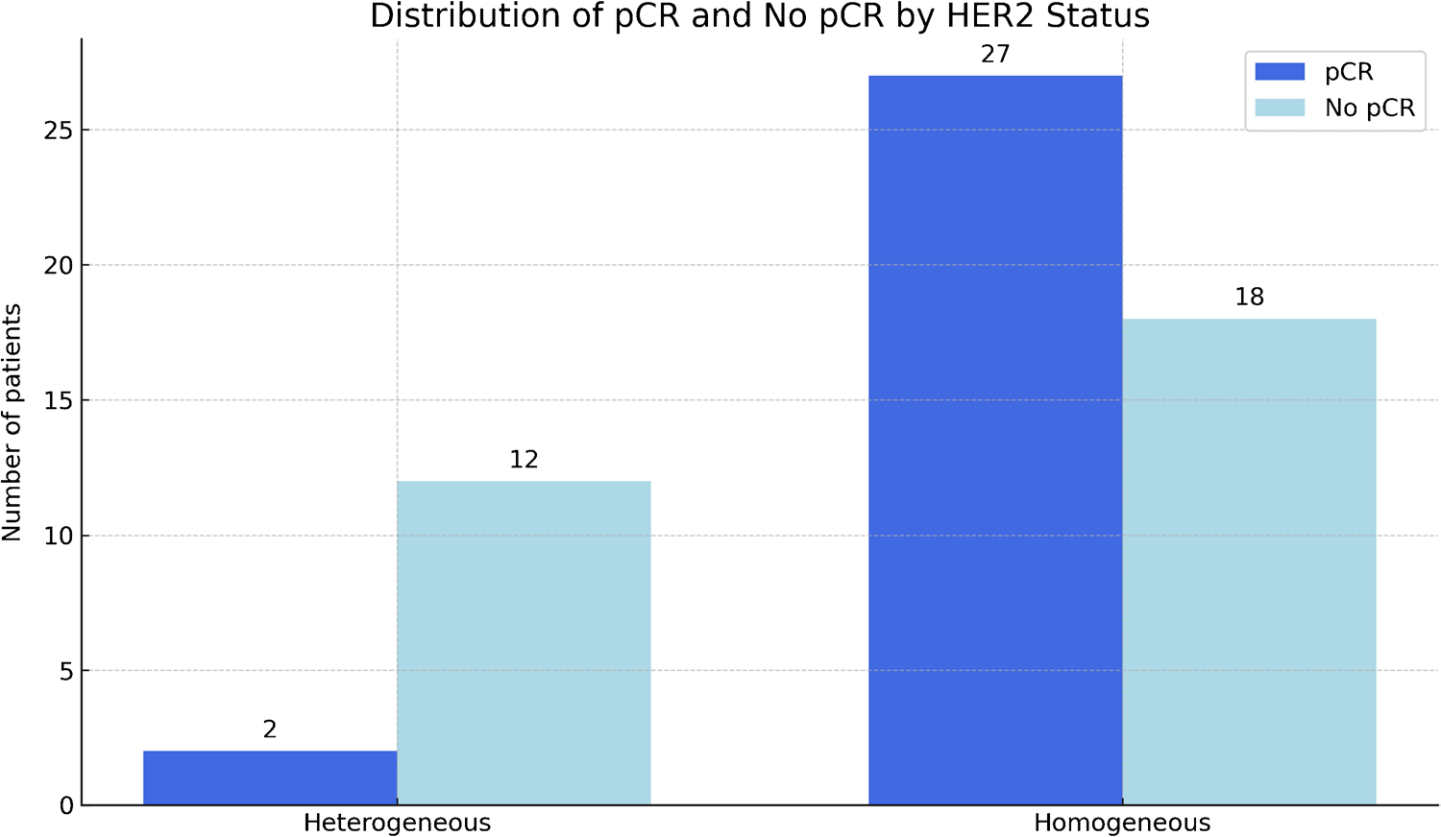
Distribution of pCR and no pCR by HER2 status. Bar chart showing the distribution of patients achieving pathological complete response (pCR) or not, stratified by presence or absence of HER2 intratumoral heterogeneity (ITH)

Clinical staging showed that 55.9% of patients were classified as cT2, while 23.7% presented with no clinically evident nodal involvement (cN0). Nearly all tumors exhibited a high proliferative index (Ki67 ≥ 20% in 96.6% of cases). Intratumoral HER2 heterogeneity (HER2 ITH) was detected in 23.7% of pre-treatment biopsies (Fig. 1). Baseline clinicopathological features are summarized in Table 1.

**Table 1:** Baseline Clinicopathological Features According to Pathological Complete Response (pCR). Comparison of demographic, histological and biological parameters between patients achieving pCR and those with residual disease

	*Total59 patients*	*pCR 29 patients (49.2%)*	*No pCR30 patients (50.8%)*	*P-value*
*Age (years)*	51.6 ± 12.3 (49.6; 43 – 60.5)	54 ± 12.2(50.4; 44.8 – 62.5)	49.3 ± 12.1(48.1; 38.1 – 58.4)	0.142
*BMI (kg/m²)*	25.5 ± 4.5 (24.7; 21.9 – 28.5)	25.2 ± 5(24.5; 21.2 – 28.5)	25.8 ± 4.0(25.1; 23.5 – 28.8)	0.611
*Histological type - IDC - ILC - Other NST*	- 49 (83.1%)- 2 (3.4%)- 8 (13.6%)	- 23 (79.3%)- 0 (0%)- 6 (20.7%)	- 26 (86.7%)- 2 (6.7%)- 2 (6.7%)	0.122
*Tumor grade - 2 - 3*	- 8 (13.6%)- 51 (86.4%)	- 5 (17.2%)- 24 (82.8%)	- 3 (10.0%)- 27 (90.0%)	0.472
*Clinical T stage - 1 - 2 - 3 - 4*	- 4 (4.8%)- 33 (55.9%)- 18 (30.5%)- 4 (6.8%)	- 3 (10.3%)- 16 (55.2%)- 9 (31.0%)- 1 (3.4%)	- 1 (3.3%)- 17 (56.7%)- 9 (30.0%)- 3 (10.0%)	0.551
*Clinical T stage (mm)*	43.4 ± 23.9 (35; 25 – 60)	43 ± 26(35; 25.5 - 60)	43.8 ± 22.1(37.5; 25 - 60)	0.903
*Clinical N stage - 0 - 1 - 2 - 3*	- 14 (23.7%)- 26 (44.1%)- 17 (28.8%)- 2 (3.4%)	- 6 (20.7%)- 13 (44.8%)- 9 (31.0%)- 1 (3.4%)	- 8 (26.7%)- 13 (43.3%)- 8 (26.7%)- 1 (3.3%)	0.943
*Estrogen receptor - 0 - <1% - 1% - 100%*	- 20 (33.9%)- 39 (66.1%)	- 13 (44.8%)- 16 (55.2%)	- 7 (23.3%)- 23 (76.7%)	0.103
*Progesterone receptor - 0 - <1% - 1% - 100%*	- 28 (47.5%)- 31 (52.5%)	- 18 (62.1%)- 11 (37.9%)	- 10 (33.3%)- 20 (66.7%)	0.038
*Ki-67 index - <20% - ≥ 20%*	- 2 (3.4%)- 57 (96.6%)	- 1 (3.4%)- 28 (96.6%)	- 1 (3.3%)- 29 (96.7%)	1.000
*HER2 - 2+ SISH/FISH amplified - 3+*	- 11 (18.6%)- 48 (81.4%)	- 1 (3.4%)- 28 (96.6%)	- 10 (33.3%)- 20 (66.7%)	0.006
*HER2 heterogeneity in biopsy - Yes - Percentage > HER2 3+ - Percentage > HER2 2+ - No*	- 14 (23.7%) - 7 (50.0%) - 7 (50.0%)- 45 (76.3%)	- 2 (6.9%) - 1 (50.0%) - 1 (50.0%)- 27 (93.1%)	- 12 (40.0%) - 6 (50.0%) - 6 (50.0%)- 18 (60.0%)	0.005
*HER2+ tested on biopsy - Heterogeneous - Percentage > HER2 3+ - Percentage > HER2 2+ - Homogeneous*	12 (%)- 9 (%) - 2 (%) - 7 (%)- 3 (%)	1 (3.4%)- 1 (100%) - 0 (0.0%) - 1 (100%)- 0 (0.0%)	11 (%)- 8 (%) - 2 (%) - 6 (%)- 3 (%)	-
*Biological subtype - ER/PgR neg - ER/PgR pos - ER/PgR low*	- 18 (30.5%)- 35 (59.3%)- 6 (10.2%)	- 12 (41.4%)- 13 (44.8%)- 4 (13.8%)	- 6 (20.0%)- 22 (73.3%)- 2 (6.7%)	0.078

### Pathological response to neoadjuvant therapy

Overall, 29 patients (49.2%) achieved pathological complete response (pCR), defined as ypT0/is ypN0, while 30 patients (50.8%) exhibited residual invasive disease after neoadjuvant therapy.

**Fig. 2:**
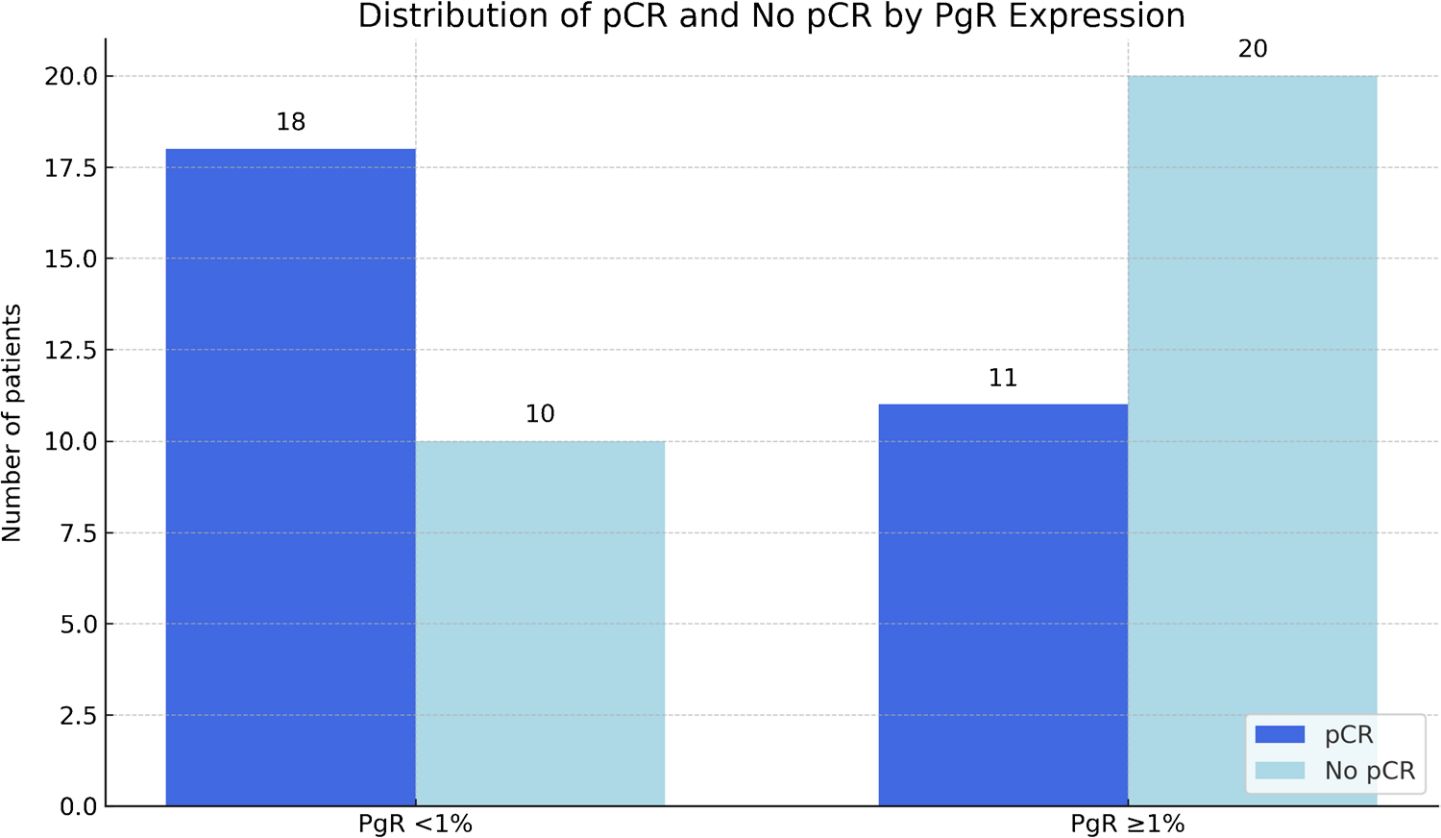
Distribution of pCR and no pCR by PgR expression. Bar chart illustrating the relationship between PgR expression levels and achievement of pCR. Patients with PgR < 1% had higher rates of pCR

In univariate analysis, pCR was significantly associated with HER2 IHC score (*p* = 0.006), HER2 ITH (*p* = 0.005), and PgR negativity (*p* = 0.038) (Fig. 2). No significant differences were observed for age, BMI, histological subtype, tumor grade, clinical tumor size (cT), clinical nodal status (cN), or Ki67 index.

**Fig. 3:**
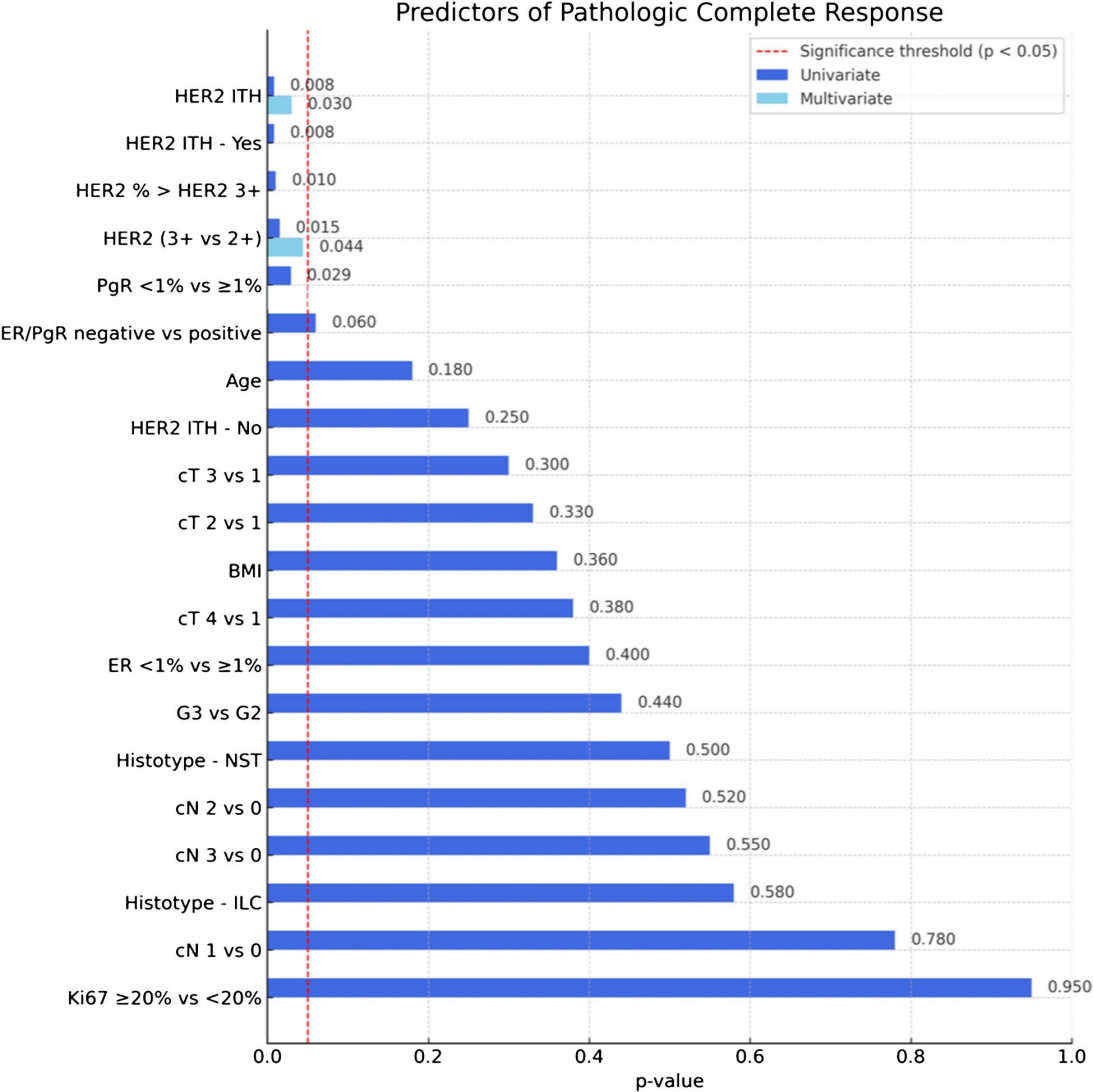
Predictors of pathologic complete response. Horizontal bar plot showing p-values from univariate and multivariate analyses of variables associated with pCR. Red dashed line indicates the threshold of statistical significance (*p* = 0.05)

Multivariate logistic regression analysis confirmed HER2 ITH as an independent negative predictor of pCR (OR 0.156; 95% CI: 0.029–0.838; *p* = 0.030), together with HER2 3 + score (OR 9.630; 95% CI: 1.065–87.085; *p* = 0.044) and PgR negativity (OR 0.306; 95% CI: 0.105–0.888; *p* = 0.029) (Fig. 3). Indipendent predictors of pathological complete response identified by univariate and multivariate analysis are reported in Table 2.

**Table 2:** Predictors of Pathologic Complete Response: Univariate and Multivariate Analysis. Odds ratios (OR), confidence intervals (CI) and p-values for variables evaluated in univariate and multivariate logistic regression

	Univariate analysis			Multivariate analysis		
	OR	P-value	95% CI	OR	P-value	95% CI
*Age (years)*	1.033	0.143	0.989 – 1.079			
*BMI (kg/m²)*	0.970	0.605	0.864 – 1.089			
*Histological type- IDC- ILC- Other NST*	0.2950.000Ref.	0.3680.1580.999	0.054 – 1.607 0			
*Tumor grade - 2- 3 *	Ref. 0.533	0.4220.422	0.115 – 2.471			
*Clinical T stage- 1- 2 - 3 - 4*	Ref. 0.3140.3330.111	0.6100.3360.3780.178	0.030 – 3.3380.029 – 3.8420.005 – 2.727			
*Clinical N stage- 0- 1- 2- 3*	Ref. 1.3331.5001.333	0.9550.6660.5770.849	0.360 – 4.9330.361 – 6.2300.069 – 25.912			
*Estrogen receptor- 0 - <1%- 1% - 100%*	Ref.0.375	0.0850.085	0.122 – 1.146			
*Progesterone receptor- 0 - <1%- 1% - 100%*	Ref.0.306	0.0290.029	0.105 – 0.888			
*Ki-67 index- <20%- ≥ 20%*	Ref.0.966	0.9810.981	0.058 – 16.199			
*HER2- 2+ SISH/FISH amplified- 3+*	Ref.14.000	0.0150.015	1.657 – 118.306	Ref.9.630	0.0440.044	1.065 – 87.085
*HER2 heterogeneity in biopsy- Yes- No*	Ref.0.111	0.0080.008	0.022 – 0.557	Ref.0.156	0.0300.030	0.029 – 0.838
*HER2 heterogeneity in biopsy - Percentage > HER2 3+ - Percentage > HER2 2+*	Ref.0.143	0.0810.081	0.016 – 1.271			
*Biological subtype- ER/PgR neg - ER/PgR pos- ER/PgR low *	Ref.0.2951.000	0.0910.0461.000	0.089 – 0.9770.141 – 7.099

### HER3 expression and association with response

HER3 immunohistochemical expression was evaluated on pre-treatment biopsies. Although a non-significant trend was observed, HER3 expression (categorized as negative, low, or high) did not significantly correlate with the achievement of pCR (p > 0.05). However, HER3-high tumors showed a consistent trend toward lower pCR and shorter EFS, aligning with preclinical data.

### Surgical outcomes and characteristics of residual disease

The types of breast surgery performed included quadrantectomy in 55.9% of cases, level II oncoplastic surgery in 13.6%, conservative mastectomy in 28.8%, and radical mastectomy in 1.7%. Axillary surgery consisted of sentinel lymph node biopsy in 42.4% and axillary dissection in 57.6% of patients. Among the 30 patients without pCR, residual tumors were predominantly classified as ypT1 (80.0%), with ypN0 nodal status in 56.7% of cases. Reevaluation of residual tumors showed that 23.3% had ER loss (ER < 1%), and 36.7% showed PgR loss. HER2 status was maintained in most cases, although 36.7% of non-pCR tumors exhibited HER2 2 + amplification rather than 3 + expression. HER2 ITH was observed in 30% of surgical specimens with residual disease. Details of surgical treatment and histopathological findings are provided in Table 3.

**Table 3:** Surgical Treatment and Histopathological Evaluation After Neoadjuvant Therapy. Type of surgery and findings on post-treatment specimens in the whole cohort and in the subset of patients without pC

Number of cases	59
*Type of breast surgery- Quadrantectomy- Level II oncoplastic surgery- Conservative mastectomy- Radical mastectomy*	- 33 (55.9%)- 8 (13.6%)- 17 (28.8%)- 1 (1.7%)
*Type of axillary surgery- Sentinel lymph node biopsy- Axillary dissection*	- 25 (42.4%)- 34 (57.6%)
*pCR- Yes- No*	- 29 (49.2%)- 30 (50.8%)
Assessment in 30 non-pCR patients	
*ypT stage- 0- 1- 2*	- 2 (6.7%)- 24 (80.0%)- 4 (13.3%)
*ypN stage- 0- 1- 2- 3*	- 17 (56.7%)- 10 (33.3%)- 2 (6.7%)- 1 (3.3%)
*Estrogen receptor re-evaluation- 0 - <1%- 1% - 100%*	- 7 (23.3%)- 14 (46.7%)
*Progesterone receptor re-evaluation- 0 - <1%- 1% - 100%*	- 11 (36.7%)- 10 (33.3%)
*Ki-67 index re-evaluation- <20%- ≥ 20%*	- 10 (33.3%)- 11 (36.7%)
*HER2 re-evaluation- 2+ SISH amplified- 3+*	- 11 (36.7%)- 9 (30%)
*HER2 heterogeneity in surgical specimen- Yes - Percentage > HER2 3+ - Percentage > HER2 2+- No*	- 9 (30.0%) - 3 (33.3%) - 6 (66.7%) - 12 (40%)

### Survival outcomes

After a median follow-up of 68.8 ± 14 months, a total of 13 events were recorded, including local–regional recurrences, distant metastases, and deaths from cancer-related and unrelated causes.

**Fig. 4:**
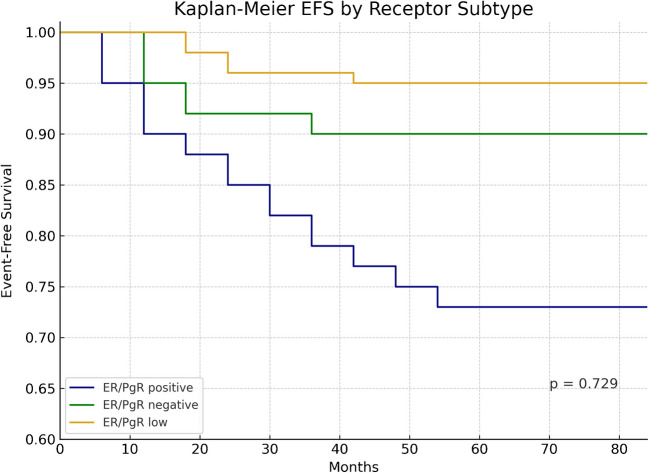
Kaplan–Meier EFS by receptor subtype. Kaplan–Meier curves for event-free survival (EFS) stratified by hormone receptor status (ER/PgR positive, negative, or low). No statistically significant differences were observed (*p* = 0.729)

**Fig. 5:**
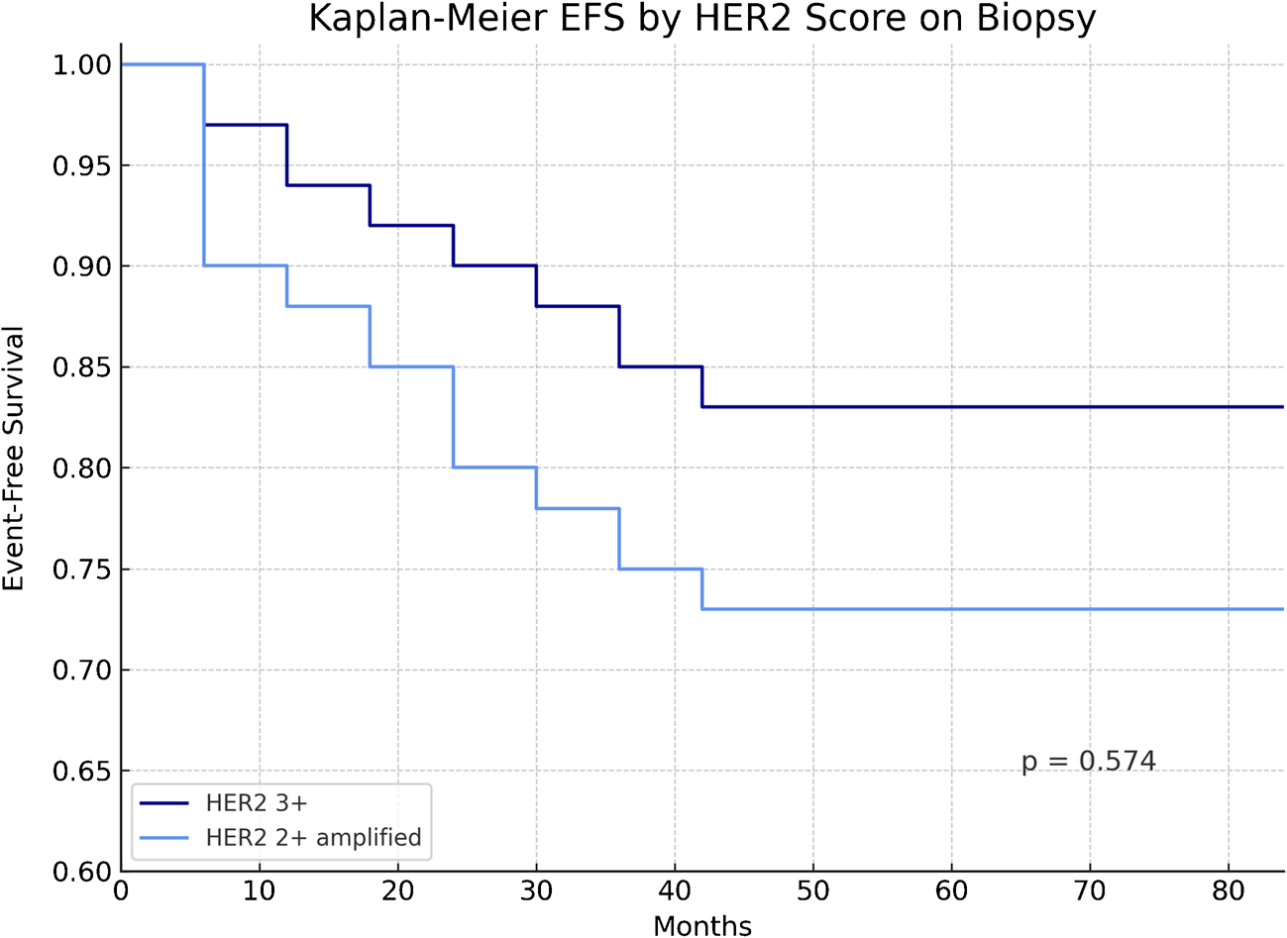
Kaplan–Meier EFS by HER2 score on biopsy. Kaplan–Meier curves for EFS comparing patients with HER2 IHC score 3 + versus HER2 2 + with amplification. No statistically significant difference was detected (*p* = 0.574)

**Fig. 6:**
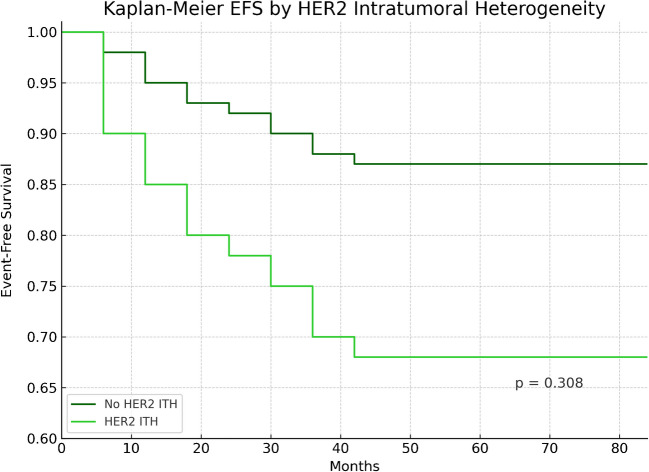
Kaplan–Meier EFS by HER2 intratumoral heterogeneity. EFS curves stratified by presence of HER2 ITH on pre-treatment biopsy. No significant difference was observed between groups (*p* = 0.308)

The overall survival (OS) rate was 83.9%, and the event-free survival (EFS) rate was 76.7%. Kaplan–Meier survival curves demonstrated no statistically significant differences in EFS according to hormone receptor status (*p* = 0.729) (Fig. 4), HER2 score (*p* = 0.574) (Fig. 5), or HER2 ITH (*p* = 0.131) (Fig. 6).

**Fig. 7:**
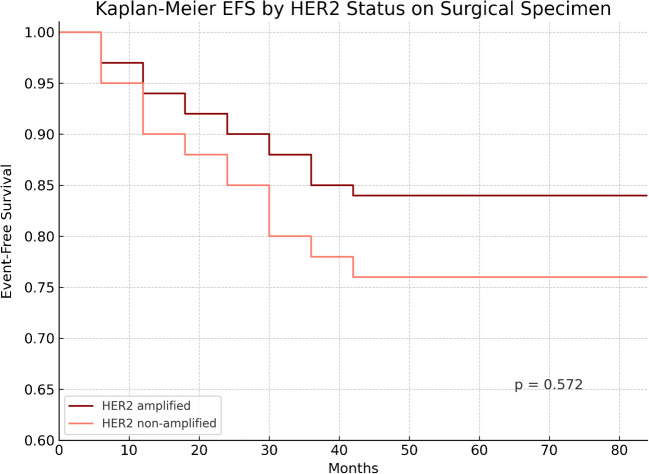
Kaplan–Meier EFS by HER2 status on surgical specimen. EFS curves for patients without pCR stratified by HER2 status in surgical specimens after neoadjuvant therapy. No statistically significant association was found (*p* = 0.572)

**Fig. 8:**
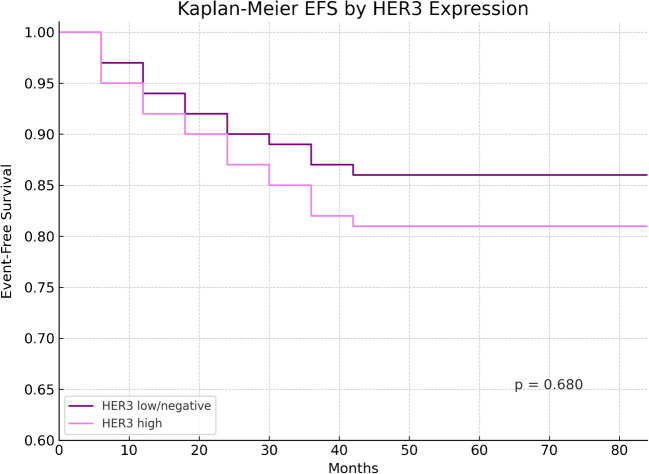
Kaplan–Meier EFS by HER3 expression. Kaplan–Meier curves showing EFS based on HER3 expression levels. High HER3 expression was not significantly associated with EFS (*p* = 0.680)

Among patients who failed to achieve pCR, HER2 status on surgical specimens did not significantly impact EFS (*p* = 0.598) (Fig. 7). Similarly, HER3 expression categories were not significantly associated with EFS (*p* = 0.680) (Fig. 8), although HER3-high expression tended to correlate with reduced survival. In fact, among HER3-high tumors, 3 of 7 patients (42.9%) experienced an event, compared to 10 of 52 (19.2%) in the HER3-low/negative group.

### Summary

In summary, the achievement of pCR was significantly influenced by HER2 score, PgR status, and the presence of HER2 ITH. HER3 expression was not significantly associated with therapeutic response or survival outcomes in this cohort but remains a potential factor for future investigation.

## Discussion

Intratumoral heterogeneity (ITH) is defined as the coexistence of tumoral cells subpopulations, being different genetically, phenotypically or behaviorally within a primary tumor or between a primary tumor and its metastases. On this wave HER2 ITH is defined as the co-existence of at least two distinct tumoral cell clones with different HER2 statuses (proteic and genetic) within the same tumor. Regarding ITH, the 2018 ASCO / CAP guideline for Breast Cancer reported that unusual patterns of HER2 expression can be observed, including strong and complete staining in less than 10% of tumor cells, being suggestive of HER2 ITH [12].

HER2 ITH has been reported in up to 40% of breast cancer and characteristically associated with poor prognosis, shorter disease-free survival, decreased overall survival and less response to traditional anti-HER2 targeted therapy [6]. HER2 ITH can be classified into genetic and non-genetic categories, based on HER2 gene amplification and HER2 protein expression in same tumor cells. Three distinct patterns of genetic HER2 ITH have been described, based on the geographic (spatial) distribution of the ITH: (1) clustered (regional) type, less common, as two distinct areas with different HER2 gene amplified tumor cell populations; (2) mosaic (intermixed) type, as diffuse intermingling of cells with different HER2 gene amplification status; (3) scattered type, as isolated HER2 amplified tumor cells in a predominantly non-amplified tumor [13]. Non-genetic ITH are characterized by tumor cells with HER2 gene amplification without HER2 protein expression intermixed with tumor cells with concordant HER2 amplification and protein expression [13]. 

In this retrospective study, we evaluated the predictive and prognostic roles of HER2 intratumoral heterogeneity (ITH) and HER3 expression in HER2-positive breast cancer patients undergoing neoadjuvant chemotherapy combined with HER2-targeted agents. Our results demonstrate that HER2 ITH is significantly associated with lower pathological complete response (pCR) rates and with a trend of reduced event-free survival (EFS), even if it has not reached the statistical significance, supporting its potential as a biomarker of therapeutic resistance. In contrast, HER3 expression was not significantly associated with pCR or EFS, although high expression tended to correlate with worse outcomes, suggesting a role in resistance mechanisms that warrants further investigation.

Unlike most prior studies, our analysis included a real-world cohort uniformly treated with anthracycline- and taxane-based neoadjuvant regimens and anti-HER2 target therapy, allowing for a robust evaluation of predictive biomarkers in a standardized clinical setting. The observed impact of HER2 ITH is consistent with previous findings: Hou et al. reported that HER2 heterogeneity was independently associated with in-complete response to anti-HER2 neoadjuvant therapy [6], and Tanei et al. identified high HER2 ITH as a poor prognostic factor in HER2-positive breast cancer [14]. HER2 heterogeneity may lead to therapeutic failure by allowing HER2-negative subclones to persist and escape treatment, especially in the context of monoclonal antibodies or antibody–drug conjugates (ADCs) [15].

In particular, recent studies have shown that HER2 ITH negatively affects the efficacy of ADCs such as trastuzumab emtansine (T-DM1) and trastuzumab deruxtecan (T-DXd), which require consistent HER2 expression across the tumor to deliver cytotoxic payload effectively [16, 17]. This is especially relevant in the era of HER2-low reclassification, where precise and reproducible HER2 assessment becomes clinically critical [18]. Thus, identifying HER2 ITH pre-treatment could help stratify patients for intensified or alter-native therapeutic strategies, including ADCs or clinical trial enrollment.

Beyond HER2 ITH and HER3 co-expression, other biomarkers have also been associated with differential response to anti-HER2 therapy. In particular, the HER2/CEP17 ratio has been proposed as a quantitative indicator of amplification intensity, correlating with both trastuzumab sensitivity and overall prognosis [19]. Moreover, tumor-infiltrating lymphocytes (TILs) reflect host immune activation and have shown an independent association with pathological complete response and survival in HER2-positive breast cancer [20, 21]. Integrating HER2 ITH with these genomic and immune biomarkers may therefore enhance predictive accuracy and improve patient stratification.

HER3 (ERBB3), a kinase-impaired member of the EGFR family, contributes to resistance mechanisms by forming heterodimers with HER2 and activating downstream PI3K/AKT signaling. In breast cancer, HER3 is overexpressed in about 50–70% of all subtypes, with the HR + /HER2- molecular group with the highest expression (followed in order by HR + /HER2 + and HR-/HER2 + subtypes) [22].

In basal-like/triple-negative breast cancer is relatively low, but when present, as in the other subtypes, it has been associated with worse prognosis, metastases development, and escape from anti-tumor treatments [23]. HER3 upregulation has been related to acquired resistance to chemotherapy, hormonal therapy, and molecular HER2- and PI3K/AKT/mTOR targeting therapy [24]. Xia et al. demonstrated that HER3 activation through an autocrine heregulin loop mediates resistance to lapatinib [9], and Huang et al. showed that HER3 collaborates with IGF1R and HER2 to promote trastuzumab resistance [8]. Nonetheless HER3 expression did not significantly predict pCR or EFS in our cohort, a trend toward worse survival in HER3-high tumors suggests a possible biological role.

However, even though targeting HER3 appears to be a promising innovative approach in breast cancer, but several anti-HER3 targeted therapies, generally based on mono- and bi-specific antibodies, failed to show substantial clinical impact [25].

Only the anti HER3 antibody drug conjugates seem to have the most promising results, being active not only in the targeted cancer cell as well as for the bystander effect, also in the neighboring cells regardless of their expression of HER3. HER3-DXd demonstrated significant clinical activity across a broad range of HER3 membrane expression, both in metastatic and early breast cancer [26].

Up to date it is hard to define the clinical activity of HER3-DXd in HER3-low or HER3-negative tumors. Meantime, no validated assays exist for scoring HER3 membrane and / or intracellular expression and defining a minimal threshold for predicting the efficacy of HER3-targeting therapies. Moreover, considering the highly dynamic HER3 expression, it is still unclear in which time point of the natural history of a metastatic breast cancer, is optimal to test HER3 expression and whether the temporo-spatial HER3 dynamism could be better monitored by innovative-less invasive technologies such as circulating tumor cells (CTCs) [27].

However, although recent clinical trials support the therapeutic relevance of HER3. until now no treatment specifically targeting HER3 has been approved for clinical use in breast cancer. Patritumab deruxtecan, a HER3-directed ADC, has demonstrated promising efficacy in metastatic HER3-expressing breast cancer, regardless of HER2 status [10]. While the predictive value of HER3 IHC alone remains uncertain, integration of IHC with RNA-based quantification or genomic data (e.g., ERBB3 expression, PI3K pathway mutations) may refine its use as a biomarker. The promising results of patritumab–deruxtecan are opening the way to a new therapeutic scenario, keeping in mind possible cross resistance [28].

Finally, future directions include combining protein-based assays with next-generation sequencing or transcriptomic platforms to better capture tumor heterogeneity and resistance profiles. In particular, spatial transcriptomics and digital pathology may enable more accurate mapping of HER2 and HER3 expression within tumors, overcoming current limitations of sampling bias.

Our study has strengths, including a well-annotated cohort with long-term follow-up and standardized pathological review of both pre- and post-treatment samples. Nonetheless, limitations must be acknowledged. The retrospective and single-institution design limits generalizability. The sample size, though adequate for the primary endpoints, may lack power for subgroup analyses, particularly for HER3. Furthermore, HER3 testing, not being routine in our laboratory, restricted the number of evaluable cases. Future studies will be needed to expand and validate these findings. We believe that with a larger sample size, even the currently non-definitive results could strengthen the evidence that heterogeneity and HER3 may serve as reliable markers of response to chemotherapy and survival.

Functional validation of HER3-driven resistance was beyond the scope of this study and should be pursued in preclinical models. Emerging tools such as digital pathology, spatial transcriptomics, and AI-assisted scoring may enable more precise detection of HER2 ITH and HER3 expression, improving risk stratification and treatment personalization. In this context, the potential of automated and AI-assisted image analysis is rapidly emerging as a promising approach to standardize biomarker quantification. Deep learning–based segmentation algorithms now allow pixel-level evaluation of HER2 expression, automatically excluding non-invasive components and generating interpretable spatial maps that reflect intratumoral heterogeneity. Such approaches, recently validated for HER2 scoring in whole-slide images [29], may substantially reduce interobserver variability and complement pathologist-driven assessment in future HER2 and HER3 studies.

In conclusion, HER2 intratumoral heterogeneity emerges as a strong predictor of poor pathological response and potentially reduced survival in HER2-positive breast cancer patients undergoing neoadjuvant therapy. HER3 expression did not reach statistical significance but may contribute to resistance in selected subgroups. Together, these markers could help personalize therapy and guide the use of emerging agents, particularly ADCs and HER3-targeted therapies. Further validation in larger, multicenter cohorts is warranted.

## Contributions

Conceptualization: A.S., A.M. and G.F.Z.; A.S., F.V. and A.d. performed development of methodology and writing; A.S., A.d., A.M., E.D.R. and G.F.Z. performed review and revision of the paper; F.V. and A.S., provided acquisition, analysis and interpretation of data, and statistical analysis; F.A., G.A., An.F. and Al.F., provided technical and material support. All authors read and approved the final paper.

## Data Availability

The datasets used and/or analyzed during the current study are available from the corresponding author on reasonable request.

## Declarations

This study was conducted in accordance with Good Clinical Practice guidelines andthe Declaration of Helsinki (1975, revised in 2013). The clinical information had been retrieved from the patient’s medical records and pathology reports. Patients’ initials or other personal identifiers did not appear in any image. Finally, all samples were anonymized before histology and immunohistochemistry. Due to the retrospective nature of the study, our institution did not deem the approval of an ethical committee as necessary because the analyzed data were collected as part of diagnosis, and patients were diagnosed and treated according to national/international guidelines and agreements. Our analysis looked retrospectively at the treated patient outcomes. This was achieved internally as part of an audit/evaluation to improve our quality of care.
